# Primary Oral Mixed Neuroendocrine–Non-neuroendocrine Neoplasm (MiNEN): A Rare Case Report and Review of the Literature

**DOI:** 10.1007/s12105-024-01613-w

**Published:** 2024-02-23

**Authors:** Pawat Sripodok, Atsumu Kouketsu, Kanako Kuroda, Hitoshi Miyashita, Tsuyoshi Sugiura, Hiroyuki Kumamoto

**Affiliations:** 1 Division of Oral and Maxillofacial Oncology and Surgical Sciences, Department of Disease Management Dentistry, Tohoku University Graduate School of Dentistry, Miyagi, Japan; 2 Department of Oral and Maxillofacial Pathology, Faculty of Dentistry, Mahidol University, Bangkok, Thailand; 3 Department of Dentistry and Oral Surgery, Tohoku Medical and Pharmaceutical University Hospital, Miyagi, Japan; 4 Division of Oral Pathology, Department of Disease Management Dentistry, Tohoku University Graduate School of Dentistry, Miyagi, Japan

**Keywords:** Head and neck, MiNEN, Neuroendocrine carcinoma, Oral

## Abstract

Mixed neuroendocrine–non-neuroendocrine neoplasms (MiNENs) are rare tumors recently characterized by the presence of both neuroendocrine and non-neuroendocrine components within the same tumor tissue. Although MiNEN found their place in the WHO classification for various organs, this composite tumor in the head and neck region remains exceptionally rare. We present a case of primary oral MiNEN in a 64-year-old male located on the left side of lower gingiva. Biopsy raised suspicion of neuroendocrine carcinoma (NEC) and the patient underwent partial mandibulectomy. The resected specimen showed two distinct components of NEC and squamous cell carcinoma (SCC) with the confirmation of immunohistochemical markers. There has been no sign of recurrence nor metastasis 6 years after the surgery. In addition, we have conducted a review of published cases with potential relevance to this entity, resulting in five cases. The diverse terminology reinforces the need for a standardized classification system of oral/head and neck MiNENs.

## Introduction

Neuroendocrine neoplasms (NENs) represent a diverse group of tumors characterized by their neuroendocrine differentiation. The latest 2022 World Health Organization (WHO) classification of head and neck tumors has introduced significant updates regarding the classification of NENs, specifically focusing on the classification criteria [1]. These updates are based on variables such as mitotic count, Ki-67 proliferation index, and the presence of necrosis. By incorporating these factors, epithelial NENs have been categorized into two groups: well-differentiated NEN, also known as neuroendocrine tumor (NET), and poorly differentiated NEN, referred to as neuroendocrine carcinoma (NEC), which consists of small and large cell subtypes.

The coexistence of both NEN and non-NEN elements within a single tumor is a rare phenomenon. This composite entity have documented in mainly gastro-entero-pancreatic tract, but also other various anatomical locations including pituitary, thyroid, nasal cavity, larynx, lung, urinary system, genital organs, and skin [2]. Notably, the term *mixed neuroendocrine–non-neuroendocrine neoplasm* (MiNEN) was recently introduced by WHO in 2017 [3]. This nomenclature aimed to better encompass the diverse range of possible combinations involving non-NEN components, which contributes to the variability of morphologies, largely influenced by the tumor sites of origin [2]. It is worth mentioning that the current WHO classification for head and neck tumors does not include the classification of MiNEN yet, thus rendering the precise characterization of these entities challenging.

In this study, we reported a rare case of oral MiNEN encountered at our institution. And to enhance the understanding for this rare entity, we conducted a review and included cases from previous reports of potential cases of oral MiNEN.

## Clinical Presentation

**Fig. 1:**
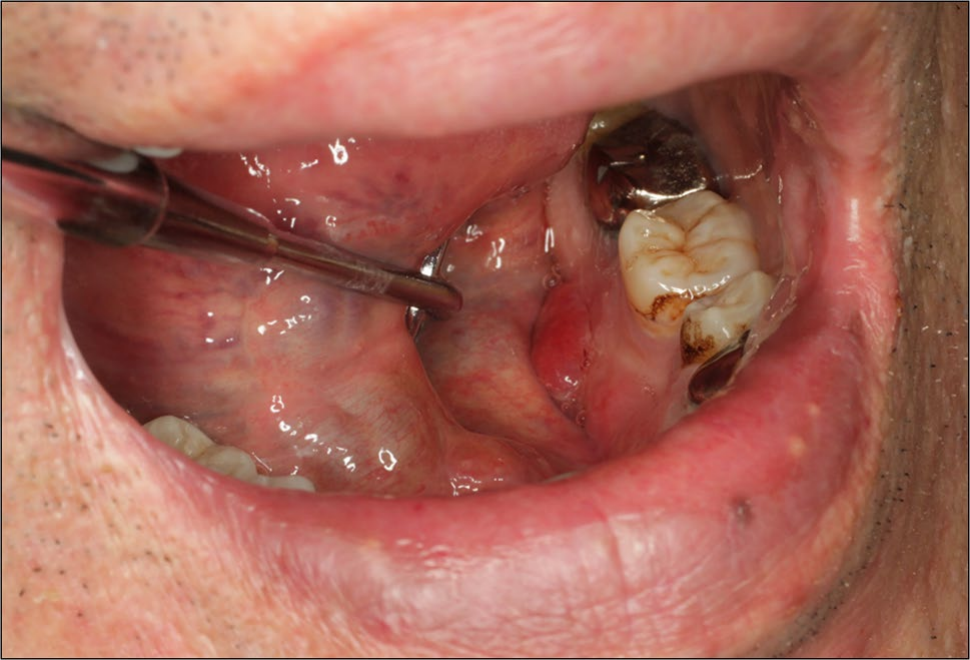
Intraoral view showing redness and swelling in the lingual site of the left mandibular molar area

A 64-year-old male patient was referred to our department on suspicion of oral carcinoma due to an enlarged swelling on the left-sided mandibular gingiva. He had a history of esophageal carcinoma and duodenal NET 4 years prior to the initial examination. A symmetrical facial feature was observed with left neck swelling. Intraoral examination revealed painless redness and swelling with a soft consistency located at the lingual gingiva of the left mandibular region, measuring about 20 × 15 mm in size (Fig. 1).

**Fig. 2:**
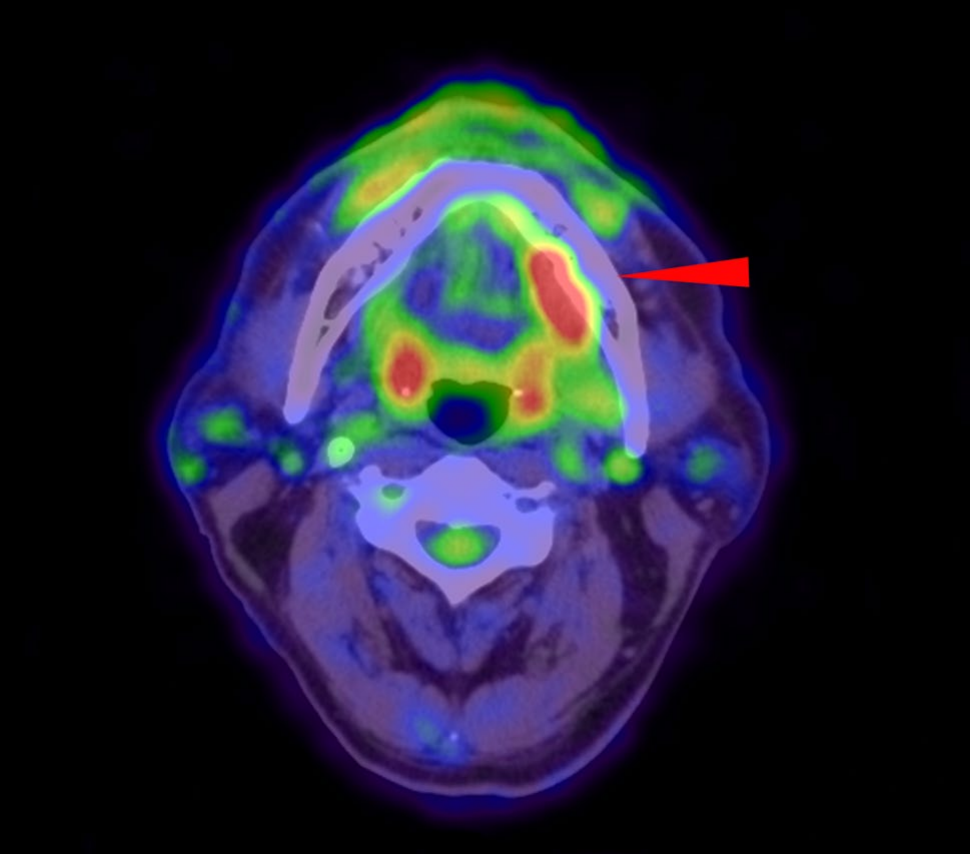
PET/CT imaging showing increased FDG uptake in the lingual side of left mandibular molar area (arrowhead)

No obvious abnormality was detected from the orthopantomogram or computed tomography (CT) scan. Fluorodeoxyglucose-positron emission tomography (FDG-PET)/CT showed aberrant FDG uptake with a boundary SUV max of 7.5 in the lingual area of the left posterior mandible (Fig. 2). No significant FDG uptake was observed anywhere other than the left mandible. The patient underwent an incisional biopsy under local anesthesia and was diagnosed as carcinoma with endocrine differentiation. Thus, a partial mandibulectomy was performed under general anesthesia.

## Pathology

**Fig. 3:**
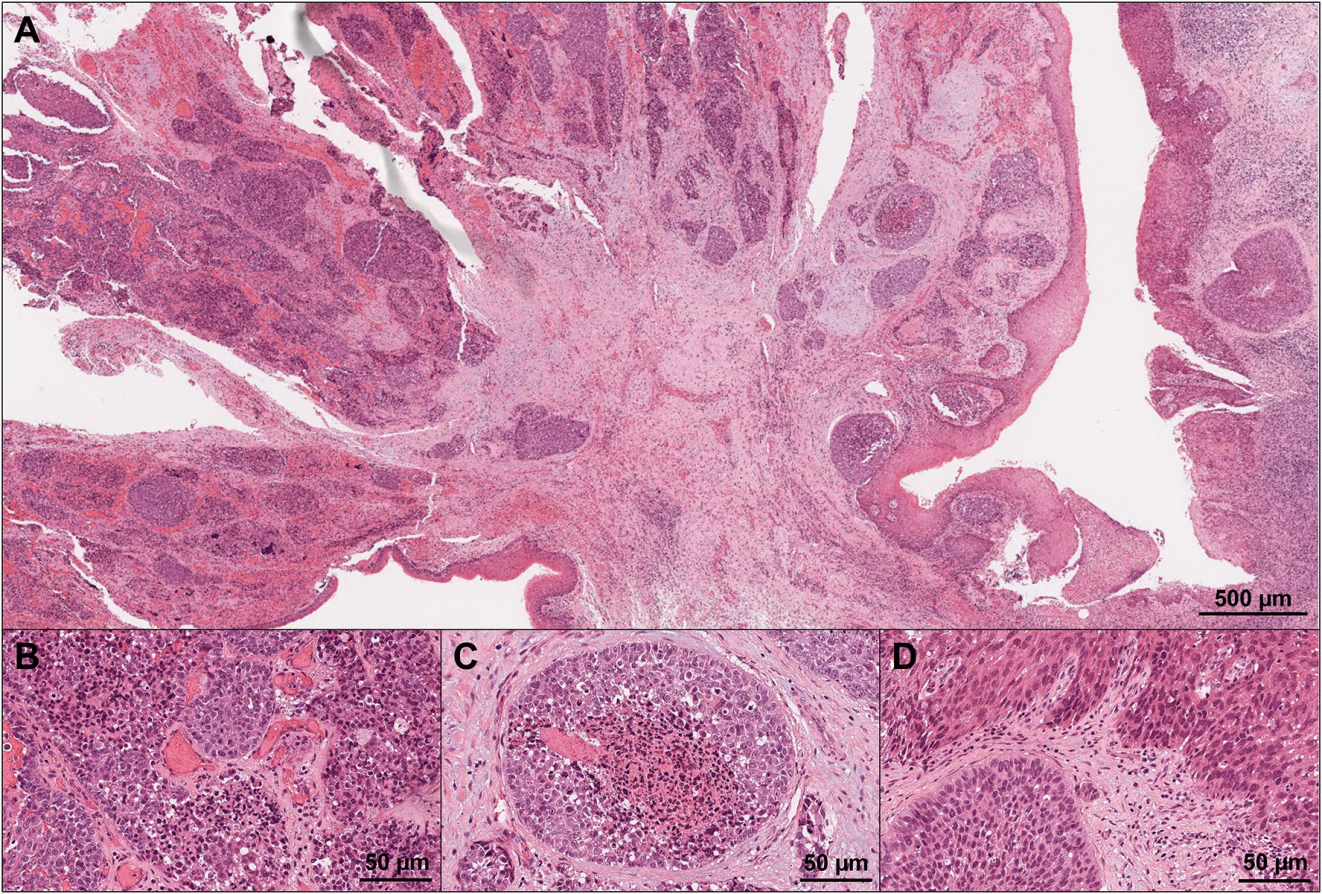
Histopathological finding from hematoxylin–eosin staining. **A** The figure containing NEC on the left, and SCC on the right, confirming oral cavity as the primary site (×40). **B** NEC showing proliferation of large-sized undifferentiated cells with eosinophilic cytoplasm and large nuclei (×400). **C** Comedonecrosis-like appearance (×400x). **D** SCC showing proliferation of atypical cells with squamous differentiation (×400)

Histological examination of the resected specimen revealed exophytic and invasive growth of neoplastic tissue along with focal ulceration (Fig. 3A). Neoplastic tissue consisted of two components: large-sized undifferentiated cell proliferation with comedonecrosis-like appearances (Fig. 3B, C), suggesting NEC and atypical cell proliferation with squamous differentiation, suggesting squamous cell carcinoma (SCC) (Fig. 3D).

**Fig. 4:**
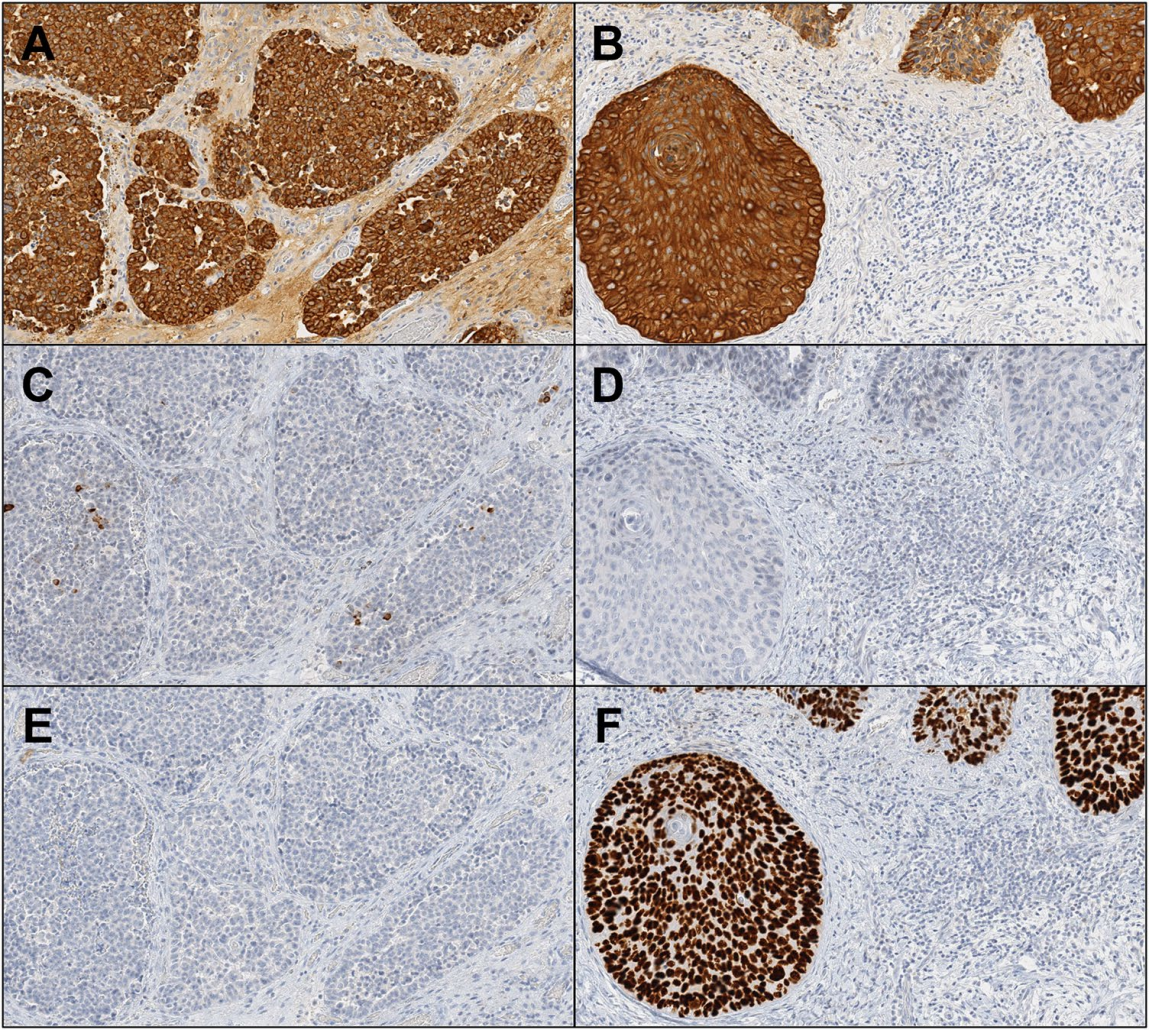
Immunohistochemical reactivity of epithelial and squamous cell markers in NEC (**A**, **C**, **E**) and SCC (**B**, **D**, **F**) (×400). (**A** and **B**: cytokeratin (AE1/3), **C** and **D**: cytokeratin 20, **E** and **F**: p40)

**Fig. 5:**
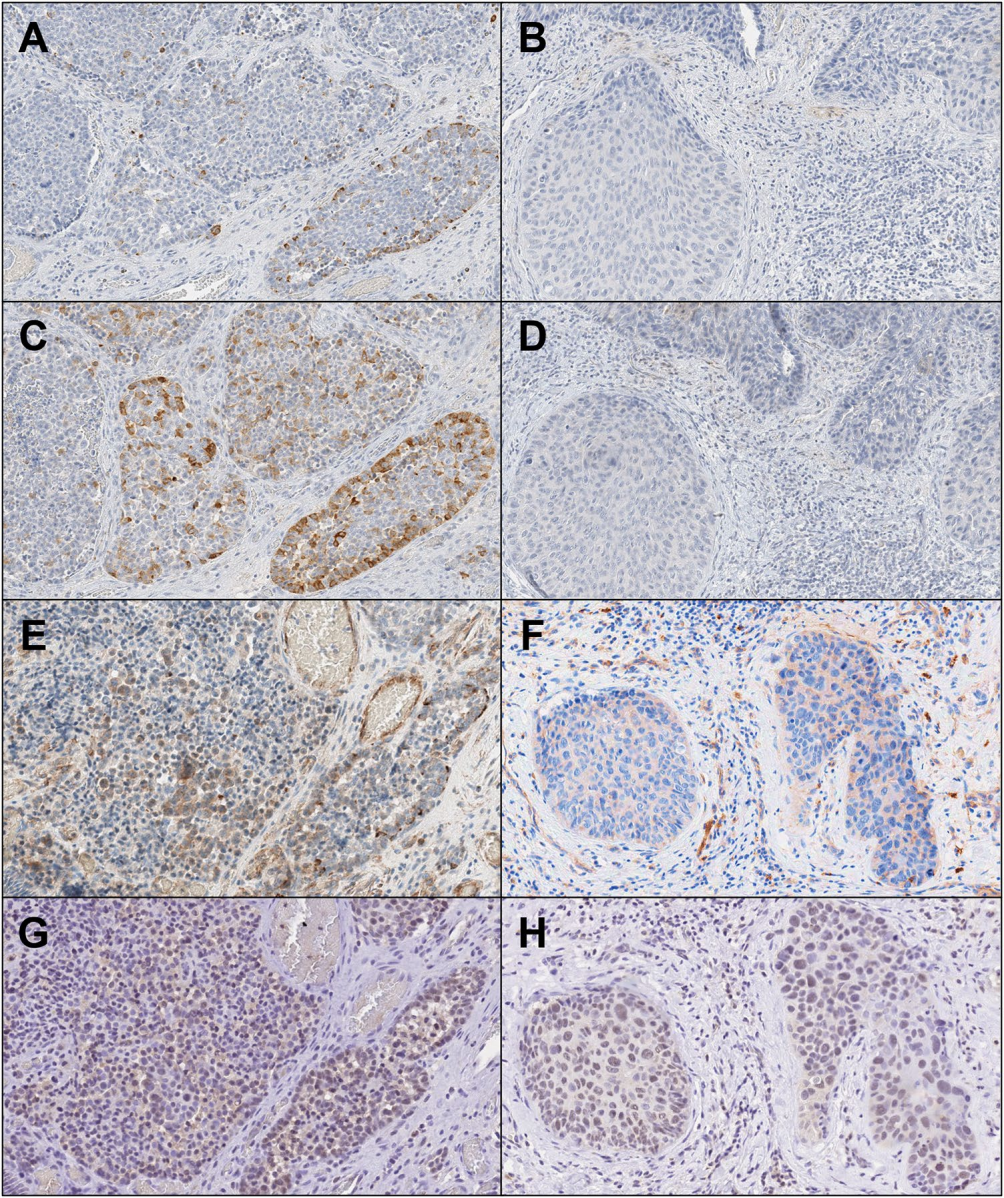
Immunohistochemical reactivity of neuroendocrine markers in NEC (**A**, **C**, **E**, **G**) and SCC (**B**, **D**, **F**, **H**) (×400). (**A** and **B**: chromogranin A, **C** and **D**: synaptophysin, **E** and **F**: CD56, **G** and **H**: INSM1)

**Fig. 6:**
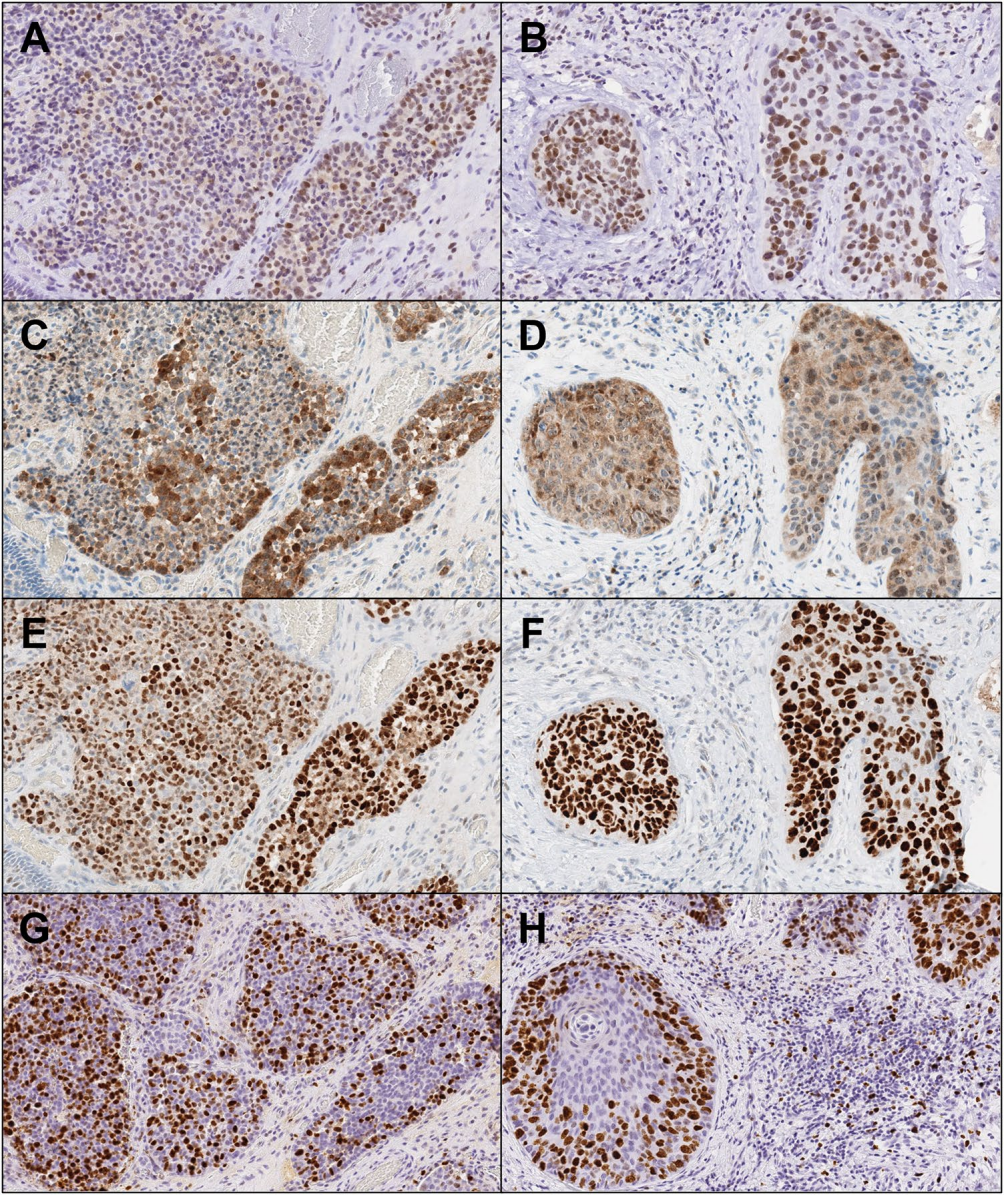
Immunohistochemical reactivity of tumor suppressor proteins and cell proliferation markers in NEC (**A**, **C**, **E**, **G**) and SCC (**B**, **D**, **F**, **H**) (×400). (**A** and **B**: Rb1, **C** and **D**: p16, **E** and **F**: p53, **G** and **H**: Ki-67)

The immunohistochemical reactivity summary is presented in Table 1. For epithelial markers (Fig. 4A–D), NEC and SCC were positive for cytokeratin (AE1/3) and negative for cytokeratin 7. NEC showed diffuse positivity toward cytokeratin (CAM5.2), while SCC was only focally positive. Cytokeratin 20 showed focal positive in NEC but not in SCC. Regard to neuroendocrine markers (Fig. 5), the NEC component exhibited focal reactivity toward chromogranin A (15% of neoplastic cells) and synaptophysin (31% of neoplastic cells), while CD56, insulinoma-associated protein 1 (INSM1), and ISL LIM homeobox 1 (ISL1) showed general positivity. The SCC component displayed negative expression for these markers. Squamous cell differentiation markers, cytokeratin 5/6, p40, and p60 were diffusely positive in SCC, but extremely limited in NEC (Fig. 4E, F) Regard to tumor suppressor proteins (Fig. 6A–F), both components revealed diffusely, generally, and focally immunoreactivity toward Rb, p16, and p53, respectively. A minimum of 2000 cells were evaluated at the hot spot for each component, revealing a Ki-67 proliferative index of 71% for the NEC element and 54.2% for the SCC segment at the identified site (Fig. 6G, H). The previous duodenal NET revealed different histopathological features, leading to the diagnosis of primary MiNEN. This composite diagnosis included large cell NEC and non-keratinizing, moderately differentiated SCC. The patient remained in good health during a 6-year follow-up.

**Table 1:** Summary of immunohistochemical analysis for each tumor component

Markers	NEC	SCC
*Epithelial markers*		
Cytokeratin [AE1/3]	+++	+++
Cytokeratin [CAM 5.2]	+++	+
Cytokeratin 7	−	−
Cytokeratin 20	+	−
*Neuroendocrine markers*		
Chromogranin A	+, 15%	−
Synaptophysin	+, 31%	−
CD56	++	−
INSM1	++	−
ISL1	++	−
*Squamous cell differentiation markers*		
Cytokeratin 5/6	+	+++
p40	−	+++
p63	+	+++
*Tumor suppressor proteins*		
Rb1	+	+
p16	++	++
p53	+++	+++
*Cell proliferation marker*		
Ki-67	71%	54.2%

+++: diffusely positive, ++: generally positive, +: focally positive, −: negative

## Literature Review

Since the introduction of MiNEN definition in approximately 2017, reviewing and categorizing these tumors has posed challenges. Prior reports have employed various terms such as *coexistence*, *collision*, *combined*, *composite*, or *mixed*, making it more challenging to gather those rare cases. In our investigation, we conducted a review using these aforementioned terms and employed a snowballing technique to identify additional references. This process ultimately yielded five cases written in English, located at the oral cavity, for inclusion in the review. Three articles originated from Japan [4–6], and one each from India [7] and the USA [8]. The relevant information including authorship, publication year, patient’s details (age, sex, site), biopsy results, diagnoses for each component, Ki-67 labeling index, treatment modalities, and patient outcomes were collected, when available. These details are summarized in Table 2.

**Table 2:** Clinical and histological features of potential oral MiNEN cases

No.	Author, year	Age (year), sex	Site	Biopsy results	NEN components	Non-NEN components	Ki-67 Labeling index	Treatment	Outcomes
1	Mochizuki et al., 2010 [4]	62, female	Upper gingiva	NEC	NEC	SCC	>80%	Partial maxillectomy	23 months, NED
2	Yamagata et al., 2016 [5]	65, male	Oral floor	NET(G2), SCC	NET(G2)	SCC	–	1st: chemoradiotherapy 2nd: tumor excision with neck dissection	1st: 6 months recurrence only SCC 2nd: 6 months metastasis to lung and mediastinum lymph nodes
3	Udompatanakorn et al., 2018 [6]	59, male	Soft palate	SCC	Small cell NEC	SCC	–	Tumor excision with neck dissection	30 months, NED
4	Bal et al., 2021 [7]	32, male	Palate	Carcinoma ex-pleomorphic adenoma, suggestive	Small cell NEC	Salivary duct carcinoma	NEC: 75%	Tumor excision with neck dissection, radiotherapy	6 months, NED
5	Archibald et al., 2022 [8]	53, male	Oral floor, retromolar trigone	Basaloid SCC	Large cell NEC	Basaloid SCC	–	Wide tumor excision^a^ with neck dissection	8 months, NED
6	Current case, 2023	64, male	Lower gingiva	NEC	Large cell NEC	Non-keratinizing SCC	NEC: 71% SCC: 54.2%	Partial mandibulectomy	72 months, NED

*NEC* neuroendocrine carcinoma, *NET* neuroendocrine tumor, *G2* grade 2, *SCC* squamous cell carcinoma, – no data available, *NED* no evidence of disease

^a^The patient underwent partial glossectomy, segmental mandibulectomy, partial pharyngectomy, tonsillectomy, and tracheostomy

Each case is uniquely located within the oral cavity. The age distribution ranged from 32 to 65 years, with an average age of 55.6 years. The male-to-female ratio was 5:1. The non-NEN elements were predominantly SCC in five cases (83.3%), while one case demonstrates salivary duct carcinoma as the non-NEN counterpart. Proliferative index data are available for only three cases (50%). One case has experienced both recurrence and metastasis. In a follow-up visit, there is no evidence of disease in five cases (83.3%), with follow-up times ranging from 6 to 72 months.

## Discussion

Traditional WHO classifications for tumors of endocrine organs and the digestive system conventionally defined MiNEN in which each component comprises at least 30% of the lesion [9, 10]. However, the threshold was set arbitrarily without concrete evidential support [2]. In certain organs, such as the esophagus, stomach, and colorectum, the WHO classification has not established a specific minimum percentage for defining MiNEN [10]. The current case has been diagnosed as MiNEN according to traditional WHO classifications. This diagnosis is supported by the fact that both NEN and non-NEN components each constitute more than 30% of the lesion. Three theories have been proposed to explain the origins of MiNENs, as the pathogenesis remains a subject of debate. The first theory suggests that NEN and non-NEN components develop from distinct cells and eventually merge. The second theory postulates that both components originate from a common pluripotent stem cell progenitor, which undergoes differential differentiation during carcinogenesis. The third theory assumes a shared monoclonal origin for both counterparts, but the NEN counterpart evolves from the non-NEN cells, driven by the progressive accumulation of genetic abnormalities [11]. The findings from our case suggested the third theory, as a part of conventional SCC was considered to obtain undifferentiated morphology and neuroendocrine properties.

Diagnosing MiNEN necessitates a comprehensive evaluation throughout the tumor tissue, given the varied possibilities in proportions of NEN and non-NEN components. Consequently, it is not infrequent to miss the diagnosis on small diagnostic biopsies, only to discover MiNENs on examination of excised samples [2]. Intriguingly, the initial biopsy was able to identify mixed histopathological features in only a third of cases [11]. To confirm the presence of NEN elements with neuroendocrine differentiation, immunohistochemical markers including synaptophysin, chromogranin A, and INSM1 are highly recommended. Although CD56 is used in specific contexts, caution is advised in its interpretation. ISL1 has also been proposed for NEN identification [9]. In our case, there was positivity for all mentioned markers, albeit with varying degrees. Additionally, immunohistochemistry for tumor suppressor proteins, including p53 and Rb1 can be useful in detecting gene abnormalities, which is common in NEC [9]. Our case revealed overexpression of p53 and focal positivity of Rb1, consistent with prior studies on NEC in the head–neck region [12]. P16 staining is typically considered evidence of HPV-related oncogenesis, but its overexpression may not be HPV-related, as Alos et al. found no HPV DNA in p16-overexpression head–neck NECs [13]. Although the precise HPV status by in situ hybridization in our case could not be analyzed, p16 immunostaining behavior was not interpreted as typical HPV-related lesion. Besides, these molecules expression did not show difference between NEC and SCC parts. In summary, our case exhibited two distinct components positive for neuroendocrine markers and marked positivity for squamous cell differentiation markers, leading to a diagnosis of MiNEN.

Given the limited number of cases available, uncertainty persists whether the prognosis of MiNEN aligns more closely with that of NEN or its non-NEN constituents. Recent review have proposed that the biological behavior of MiNENs is predominantly influenced by the NEN component, which tends to be poorly differentiated and often found in distant metastatic sites [11]. In the context of head and neck region, the cancer-specific survival (CSS) rates for patients with NEC at 5 years were reported at 43% [14], whereas CSS for patients SCC in the head and neck region stood notably higher at 63% [15]. These findings underscore that the prognosis of NEN, especially NEC, is notably poorer than that of SCC. In our case, the Ki-67% labeling index for the NEC component was 71%, whereas the SCC component showed a lower index of 54.2%. This suggests that the biological behavior of the NEN counterpart is more active than its non-NEN counterpart. Notably, patients with MiNEN faced a poorer outcome as compared to those with pure NEC in the small intestine and appendix, although there were no significant survival differences between NEC and MiNEN in other parts of the gastrointestinal system [16]. This raises the possibility that the prognosis of MiNEN may be more aggressive than NEC, although it could potentially be site specific. In the reviewed cases of oral MiNENs [4–8], a high proliferation index (over 50%) did not seem to correlate with poorer outcomes, suggesting that proliferation rate may not be the key determinant of outcomes. Also, in our case, the patient underwent surgical treatment alone and remained in good health during the 6-year follow-up period.

In addition to our presented case and the cases reviewed, there have been reports of potential MiNEN cases in head and neck, including nasal cavity, sinonasal tract, oropharynx, palatine tonsil, larynx, hypopharynx, and parathyroid [17–25]. Notably, data from these studies consistently point to SCC as the predominant non-NEN component [4–6, 8]. Nevertheless, this observation aligns with the fact that SCC is the most common cancer in the head and neck region, ranking the sixth most common cancer worldwide [26]. MiNEN associated with SCC have gained recognition across the following anatomical sites: sinonasal tract, oropharynx, larynx, lung, esophagus, cervix/vagina, and skin [9]. However, the precise characterization of MiNEN in oral and other head and neck locations remains undefined. We believe that establishing an understanding of this entity is advisable. With the absence of treatment guidelines, reported oral MiNENs typically undergo surgery, with or without adjunctive radiotherapy. While the prognosis appears favorable, uncertainties persist, prompting the need for further research to clarify its clinical course and outcomes.

## Conclusion

Our study supplements the existing literature by presenting an additional case to the five potential instances of reported oral MiNENs, underscoring the rarity of this entity in the oral cavity. In the head and neck region, non-NEN components are primarily comprised of SCC, although they may encompass other carcinoma types. The diverse terminology employed in prior reports emphasizes the imperative for standardized classification of oral/head and neck MiNENs to gain a more comprehensive understanding of this intricate entity.

## Data Availability

Not applicable.

## Declarations

### Research Involving Humans and Animals

No patient’s personal data/identifiers are disclosed; hence, no consent was taken.
